# Pooled DNA sequencing in hairy vetch (*Vicia villosa* Roth) reveals QTL for seed dormancy but not pod dehiscence

**DOI:** 10.3389/fpls.2024.1384596

**Published:** 2024-04-04

**Authors:** Neal Tilhou, Lisa Kissing Kucek, Brandon Carr, Joel Douglas, John Englert, Shahjahan Ali, John Raasch, Suresh Bhamidimarri, Steven Mirsky, Maria J. Monteros, Ryan Hayes, Heathcliffe Riday

**Affiliations:** ^1^ United States (US) Dairy Forage Research Center, United States Department of Agriculture-Agricultural Research Service (USDA-ARS), Madison, WI, United States; ^2^ United States Department of Agriculture-Natural Resources Conservation Service (USDA-NRCS), James E. “Bud” Smith Plant Materials Center, Knox City, TX, United States; ^3^ United States Department of Agriculture-Natural Resources Conservation Service (USDA-NRCS), Central National Technology Support Center, Fort Worth, TX, United States; ^4^ United States Department of Agriculture-Natural Resources Conservation Service (USDA-NRCS), National Plant Materials Program, Washington, DC, United States; ^5^ Corteva Agriscience, Connell, WA, United States; ^6^ Sustainable Agricultural Systems Laboratory, United States Department of Agriculture-Agricultural Research Service (USDA-ARS), Beltsville, MD, United States; ^7^ Bayer Crop Science, North America (NA) Breeding, Chesterfield, MO, United States; ^8^ Forage Seed and Cereal Research Unit, United States Department of Agriculture-Agricultural Research Service (USDA-ARS), Corvallis, OR, United States

**Keywords:** genomic prediction, bulk sequencing, cover crop, domestication traits, genome wide association analyses

## Abstract

**Introduction:**

Hairy vetch (*Vicia villosa Roth*) is a promising legume cover crop, but its use is limited by high rates of pod dehiscence and seed dormancy.

**Methods:**

We used phenotypically contrasting pooled DNA samples (n=24 with 29-74 individuals per sample) from an ongoing cover crop breeding program across four environments (site-year combinations: Maryland 2020, Maryland 2022, Wisconsin 2021, Wisconsin 2022) to find genetic associations and genomic prediction accuracies for pod dehiscence and seed dormancy. We also combined pooled DNA sample genetic association results with the results of a prior genome-wide association study.

**Results and discussion:**

Genomic prediction resulted in positive predictive abilities for both traits between environments and with an independent dataset (0.34-0.50), but reduced predictive ability for DNA pools with divergent seed dormancy in the Maryland environments (0.07-0.15). The pooled DNA samples found six significant (false discovery rate q-value<0.01) quantitative trait loci (QTL) for seed dormancy and four significant QTL for pod dehiscence. Unfortunately, the minor alleles of the pod dehiscence QTL increased the rate of pod dehiscence and are not useful for marker-assisted selection. When combined with a prior association study, sixteen seed dormancy QTL and zero pod dehiscence QTL were significant. Combining the association studies did not increase the detection of useful QTL.

## Introduction

Winter cover crops are an important conservation strategy to reduce nutrient and soil loss (Blanco-Canqui et al., 2015). In a recent survey, non-cover crop users reported two major overlapping barriers to adoption: a lack of economic returns and potential yield reductions of cash crops following cover crops (CTIC and SARE, 2023). Currently, cereal rye *(Secale cereale* L.) is the most common cover crop by planting area, with three-fold greater use relative to the next most popular cover crop (radish, *Raphanus sativus* L.). Cereal rye is popular because it is affordable and provides reliable soil cover and weed suppression. However, cereal rye can reduce yields in subsequent cash crops through allelopathy, nitrogen immobilization, or moisture reduction (Martinez-Feria et al., 2016; Thompson et al., 2020; Wang et al., 2021). Legumes, such as hairy vetch (*Vicia villosa* Roth), could reduce some of these issues through nitrogen fixation and more rapid litter breakdown (Ranells and Wagger, 1996; Rosecrance et al., 2000; Tonitto et al., 2006; Poffenbarger et al., 2015; Marcillo and Miguez, 2017). Hairy vetch can produce large quantities of biomass, is competitive in rye mixtures, and is winter hardy in the northern United States (Ranells and Wagger, 1996; Clark et al., 1997; Hayden et al., 2014). However, hairy vetch has limited breeding history and seed is expensive relative to cereal rye (Snapp et al., 2005; Mirsky, 2017).

Two major issues with current hairy vetch cultivars are seed dormancy and pod dehiscence (shatter). These traits harm both seed producers and cover crop adopters and are major targets for breeding improved hairy vetch populations. Hairy vetch seed dormancy ranges from 0 to 100% among populations with mean dormancy of specific growing environments ranging from 4%-80% (Kissing Kucek et al., 2020a). Like other wild legumes, seed dormancy is an adaptation to unpredictable growing conditions (Renzi et al., 2014; Kissing Kucek et al., 2020a). For producers, dormant seed reduces stand density during the target growing season and hairy vetch seed can remain dormant for multiple years and results in unexpected ‘volunteer’ vetch plants in subsequent crops. Similarly, pod dehiscence is an adaptive trait for wild crops (Kissing Kucek et al., 2020b), but results in significant seed loss for seed producers and can result in ‘volunteer’ hairy vetch in subsequent cash crops. Renzi et al. (2017) reported a range of 15% to 46% pod dehiscence in Argentina across two years. There are no reports on the amount of seed loss from pod dehiscence during commercial seed production in the United States, as the rates likely vary widely due to management and environmental conditions during harvest. Reducing or eliminating pod dehiscence and dormancy, therefore, can further improve adoption of hairy vetch through reduced seed cost and reduced risk of a re-seeding hairy vetch population.

Selection for reduced pod dehiscence and seed dormancy is challenging because both traits appear quantitative and are dependent on growing conditions (Kissing Kucek et al., 2020a, Kissing Kucek et al., 2020b). A recent genome wide association analysis of a breeding panel of hairy vetch found a large-effect quantitative trait loci (QTL) for seed dormancy, but no statistically significant associations for pod dehiscence (Tilhou et al., 2023). In the Tilhou et al. (2023) dataset, it was difficult to determine if pod dehiscence was simply highly polygenic or if the field data was inadequate. The major challenge in detecting QTL associated with pod dehiscence was high dehiscence rates in the Oregon environment. The strongly left-skewed distribution (mean of 2.7 on a 0-3 visual dehiscence score) reduced statistical power and highlighted the need for further dehiscence reductions, since Oregon is a major cover crop seed production region. Further examination of dehiscence could, therefore, accelerate breeding progress for reduced pod dehiscence in hairy vetch.

To economically screen multiple environments, DNA from individuals with similar performance can be pooled and sequenced as a population (Henshall et al., 2012; Zou et al., 2016; Baller et al., 2020). In both genome-wide association studies (GWAS) and genomic prediction, this results in minimal loss of information while reducing sequencing to a fraction of the cost of individual scale genotyping (Earp et al., 2011; Yang et al., 2015; Tilhou and Casler, 2021; Tilhou et al., 2022). This study will supplement prior genetic resources with DNA pools constructed to discover associations for two traits (seed dormancy and pod dehiscence) among four growing environments (site-year combinations) of a hairy vetch breeding program (Maryland 2020 and 2022, Wisconsin 2021 and 2022). These pooled DNA samples will provide information about major genome-wide associations and will provide information about the relationship among traits across contrasting environments.

## Methods

### Field evaluation and DNA pool construction

This study sampled individuals evaluated during ongoing breeding efforts of the Cover Crop Breeding Network. Four environments were selected which had large breeding population sizes (n>100) and contained a wide range of pod dehiscence and seed dormancy values. Specifically, sites with excessively high or low pod dehiscence or seed dormancy were not used because outliers could not be identified from skewed distributions. For the remainder of this study, we will consider each site-year as distinct growing environments and refer to them using an abbreviation and final two digits of the harvest year (i.e. Beltsville, MD 2020 and 2022: 20MD, 22MD, Prairie du Sac, WI 2021 and 2022: 21WI and 22WI). Site conditions and management are summarized in **Table 1**. The 20MD breeding nursery was planted on Sept 27^th^ 2019 (39°01′50″ N, 76°55′59″ W, Russett–Christiana complex soil) into a tilled field which was broadcast seeded with turf red fescue (*Festuca rubra* L.; 44.8 g a. i. ha^-1^). On November 5^th^ 2019, 20MD was sprayed with Raptor (ammonium salt of imazamox; 0.37 L ha^−1^) to control winter weeds. For 20MD, pods were collected from June 23^rd^ 2020 to June 30^th^ 2020 (harvest varies with pod maturity of individual genotypes, see below). The 22MD nursery was planted at the same location as 20MD on October 7^th^, 2021 and pods were collected from June 29^th^ to July 5^th^ 2022. The 22MD nursery used black plastic covered raised beds to control weed pressure. The 21WI nursery was planted on September 23^rd^ 2020 (43°20′55″ N, 89°45′18″ W, Richwood silt loam soil) into landscape fabric. The 21WI nursery pods were collected from July 9^th^ to July 30^th^ 2021.The 22WI nursery was planted in early October on the same location as 21WI into landscape fabric. For 22WI, pods were collected between July 20^th^ and August 5^th^ 2022. Prior to establishment, all environments were supplemented with lime, K and P based on soil test results. Additional details on the breeding program goals and methods can be found in prior publications (Kucek et al., 2019; Kissing Kucek et al., 2020b; Tilhou et al., 2023).

**Table 1:** Management and site conditions for the four site-years used in this study.

Site-Year	Latitude	Longitude	Weed Control	Planting Date	Pod Collection Dates	Final Population Size
20MD	39°01′50″ N	76°55′59″ W	Broadcast seeded turf red fescue and herbicide*	Sept 27^th^ 2019	June 23^rd^ 2020 to June 30^th^ 2020	115
22MD	39°01′50″ N	76°55′59″ W	Black plastic covered raised beds	October 7^th^ 2021	June 29^th^ 2022 to July 5^th^ 2022	109
21WI	43°20′55″ N	89°45′18″ W	Landscape Fabric	September 23^rd^ 2020	July 9^th^ 2021 to July 30^th^ 2021	206
22WI	43°20′55″ N	89°45′18″ W	Landscape Fabric	Early October 2021	July 20^th^ to August 5^th^ 2022	287

*Raptor (ammonium salt of imazamox; 0.37 L ha^−1^; November 5^th^ 2019).

Breeding site-years consisted of direct-seeded spaced individual hairy vetch plants (20MD: n=3696; 21W1: n=1200; 22MD: n=3648; 22WI: n=1200) which were visually evaluated for fall vigor, spring vigor, and plant maturity (Kalu and Fick, 1981). Since hairy vetch is out-crossing, one round of selection based on vigor occurs in late spring prior to cross pollination (Richards, 1997). Only selected individuals are allowed to cross pollinate and are then evaluated for seed production characteristics, which includes pod dehiscence and seed dormancy. Selection intensity prior to flowering varied from 3.3 to 47% in each environment, allowing the following number of individuals to cross pollinate: 20MD: n=124, 21WI: n=560, 22MD: n=155, and 22WI: n=363.

Pods were collected for dormancy and dehiscence evaluations at ripe seed pod stage according to Kalu and Fick (1981). Pod dehiscence was estimated using the mean pod dehiscence visual score of a subsample of pods targeting a minimum of 50 pods per individual (Kissing Kucek et al., 2020b). During 2020, visual scores were on a 0-3 scale (described in Kissing Kucek et al., 2020b). Briefly, zero indicated a fully intact pod (no openings along sutures), a score of one indicated one suture was opened (one side of pod), a score of two indicted two sutures were opened (both sides of pod), and three indicated that the pod had fully opened. During 2021 and 2022, visual scores were based on a 0 or 1 scale (0: pod is closed enough that a seed could not fall out, 1: pod is open enough that a seed could fall out). For analysis, the 2020 scores were divided by three to be equivalent to the 2021 and 2022 scores. Green, flat, or immature pods were discarded prior to scoring. Seed dormancy was determined by counting the number of seeds which did not germinate. Briefly, we measured the proportion of 25 seeds which imbibed water after 7 d per individual plant, with three replicates per individual (detailed methods in Kissing Kucek et al., 2020a). Seeds which did not imbibe water after 7 d were scarified and observed after an additional 7 d to determine seed viability for environments collected in 2020. For 2021 and 2022 environments, hard seeds were determined to be viable and not scarified. Dead seed was not included in the dormant seed proportion.

Subsequent field selection for disease resistance and seed production resulted in smaller population sizes available for pod dehiscence ratings, seed dormancy ratings, and DNA pool construction (20MD: n=115; 21WI: n=206; 22MD: n=109; 22WI: n=287). Hairy vetch tissue from these individuals were collected for sequencing during active vegetative growth in mid-summer. Each leaf sample was placed into a labeled coin envelope and immediately placed on ice before transport to a laboratory freezer (-20°C) and stored until freeze drying.

For each location, trait-based pooled DNA samples were constructed from stored leaf samples using the best 25% and worst 25% performance for pod dehiscence and seed dormancy. In addition, one pooled DNA sample was constructed from the interquartile remainder for each environment by randomly sampling 25% of the remaining population (i.e. individuals included in a trait-based pooled DNA sample were not included in the random interquartile sample). Random interquartile sampled DNA pools provide estimates of the mean performance and mean allele frequency of populations for each site-year and can improve model accuracy (unpublished data). Pool construction was achieved by combining equal sized leaf tissue from each individual prior to pulverizing (Craig et al., 2009). A subsample of homogenized samples was then used to extract DNA. To help validate this method, four technical replicates were created from four randomly selected tissue samples.

### Sequencing and SNP filtering

In total, 24 DNA pools were submitted for sequencing (five pools [high pod dehiscence, low pod dehiscence, random interquartile, high seed dormancy, and low seed dormancy] by four environments [21MD, 22MD, 21WI, and 22 WI] and four technical replicates). The University of Wisconsin Biotechnology Center prepared libraries for genotype-by-sequencing using an *NsiI-BfaI* double digestion restriction enzyme digestion. Fragments were then ligated to barcoded adaptors prior to polymerase chain reaction amplification and sequencing on an Illumina sequencer (NovaSeq 6000) targeting a 20 million reads per sample (mean of approximately 15x coverage).

Bioinformatics processing was completed at the University of Wisconsin Biotechnology Center using the TASSEL analysis platform (Glaubitz et al., 2014) in parallel with re-calling single nucleotide polymorphisms (SNPs) from the Tilhou et al., 2023 GWAS panel which are hairy vetch individuals evaluated in Oregon and Texas in 2019 (n=869). Barcoded sequence read outputs were collapsed into a set of unique sequence tags with counts. These tags were aligned to the reference genome (*V. villosa* v1.1; Fuller et al., 2023). Each tag was assigned to a position with the best unique alignment, and the occupancies of tags for each sample were observed from barcode data. For pooled DNA samples, allele states were analyzed as two-times alternate allele frequencies (continuous 0-2) and individual DNA samples were analyzed as alternate allele dosages (0, 1, or 2). Overall, 2,877,384 SNPs were present prior to filtering. Of these, only 2,105,338 SNPs were mapped to the seven main chromosome fragments. Within these, 165,057 of the remainder had >30 read depths within the pooled DNA samples. This cut-off was based on the accuracy among technical replicates at varying read depths. At this point, the SNPs had 14.8% of missing values which were imputed to the mean values for the marker. Last, SNPs were removed with <0.025 minor allele frequency, resulting in 122,801 SNPs.

### Genomic prediction among experiments and environments

To validate the utility of the pooled DNA samples, the pooled DNA samples were used to train genomic prediction models and predict individual plant performance in the Tilhou et al., 2023 GWAS panel. All of these models used a genomic best linear unbiased predictor (GBLUP) model:


$$y_{jkl}=\mu +\;g_{j}+E_{k}+\epsilon_{jkl}$$

where y_jkl_ is the observed trait, μ is the overall mean, g*_j_*, is the effect of the j-th genotype, E*_k_* is the fixed effect of the *k*-th site-year combinations (environment) and ϵ*_jkl_* are residuals. For models including field data from only one environment, the environmental (E*_k_*) fixed effect is removed. For models trained using pooled DNA, the observed trait is the mean performance of the individuals included in a pooled DNA sample. For models trained using individuals from the 19OR and 19TX environments, the mean performance of each individual is included. The additive genotypic effect was estimated using marker-derived realized additive relationship as the variance-covariance matrix using the following formula: g*_j_* ~MVN(0,**A** σ_g_^2^). The **A** matrix of realized additive relationships between allele dosages of DNA pools and individuals was calculated using A.mat function in the rrBLUP package (Endelman, 2011). The residual error effects followed the formula ϵ_*j*k_ ~N(0, **D** σ_g_^2^). The **D** matrix is a diagonal matrix allowing separate error variances between environments, when more than one environment occurred in the training data. The above models were solved using the R package sommer (R Core Team; Covarrubias-Pazaran, 2016). All predictive abilities were calculated based on individual performance at the Texas (19TX) and Oregon (19OR) environments of the Tilhou et al. (2023) study. The GEBVs from a model including all individual data was used as the true breeding values. These were compared to GEBVs estimated using: (1) only 19OR individuals, (2) only 19TX individuals, (3) all DNA pools (n=24), (4) only the Wisconsin pools (21WI and 22WI, n=12), (5) only the Maryland pools (20MD and 22MD, n=12), (6) the numerically largest pooled DNA samples (22WI, n=5, 74 individuals per DNA pool), and (7) the numerically smallest pooled DNA samples (20MD, n=5, 29 individuals per DNA pool). Predictive abilities for each comparison are the correlation coefficient between predicted GEBVs and the GEBVs based on all individual field data in the model (19OR and 19TX).

### GWAS analysis

The 24 pooled DNA samples were used in two GWAS analyses for pod dehiscence and proportion of dormant seed using the mean trait values of all pooled DNA samples. The model was run using the GWAS function in the R package ‘sommer’ v.4.3.2 (Covarrubias-Pazaran, 2016). Environment effects were included as fixed effects in both models. Multiple-testing correction was based on the false discovery rate (Benjamini and Hochberg, 1995) with a significance threshold of q=0.01.

To improve the statistical power of this genome-wide association, the pool-based GWAS was combined with the individual GWAS panel previously published in Tilhou et al., 2023. To do this, a model identical to the Tilhou et al., 2023 GWAS was re-run using the SNP marker panel which was called in parallel with the pooled DNA samples. Briefly, this analysis used individual observations of pod dehiscence (n=791) and seed dormancy (n=853) across two environments. Population structure was controlled using the realized relationship matrix (K model) and the interactive effect of each maternal line in each environment was included as a random effect. The GWAS model also assumed diagonal residual covariance structure between environments.

The two sets of p-values (one from pooled samples and one from individual samples) were merged using Fisher’s combined probability test (Fisher, 1938). Prior to merging p-values, markers with effect estimates in opposite direction had the unfavorable (high seed dormancy or high pod dehiscence) test p-value set to one. Then, probabilities were merged into a test statistic (χ^2^) using


$$\chi^{2}=-2\sum_{i=1}^{k}ln\;p_{i}$$

where *p_i_* is the p-value of the *i*-th test. The χ^2^ test statistics follow a chi-squared distribution with 2k degrees of freedom, with k equal to the number of p-values (k=2 in this study).

## Results

**Figure 1:**
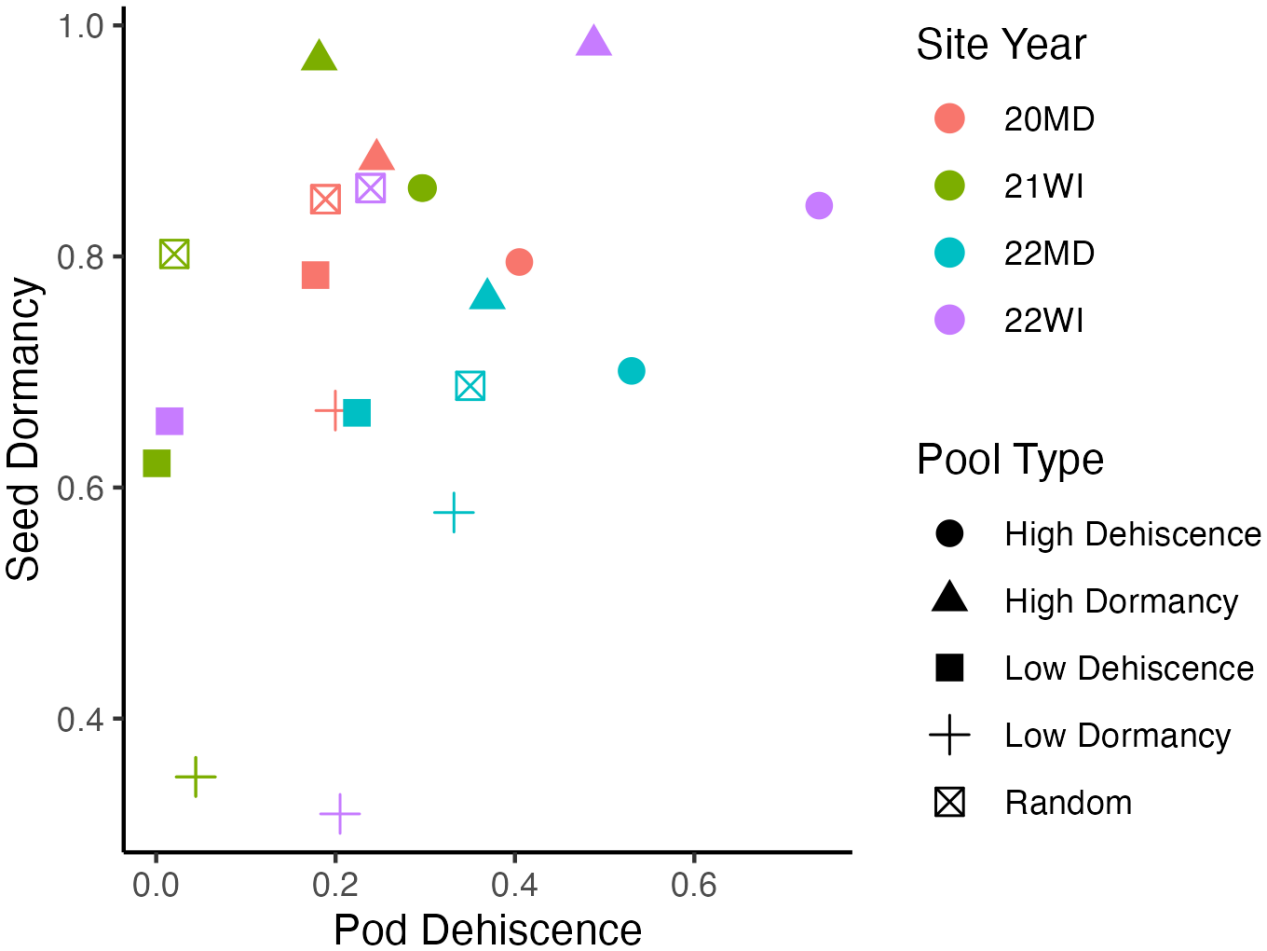
Mean phenotypic performance of individuals included in pooled DNA samples. Each point represents a pooled DNA sample. Point shapes indicate the outlier trait targeted during DNA pool construction and point colors indicate the site-year. Seed dormancy is the proportion of dormant seed. Pod dehiscence is the best linear unbiased estimate for visual pod dehiscence scores using a logistic model. Mean phenotypic correlation between the mean traits of pooled DNA samples is 0.37.

Based on technical replicates, allele frequency estimates became steadily more accurate with increasing read depth, with a plateau occurring around 30 reads per allele (**Supplementary Figure 1**). The was a positive correlation between pod dehiscence and seed dormancy based on the mean phenotypic performance of the pooled DNA samples (0.37; **Figure 1**).

**Figure 2:**
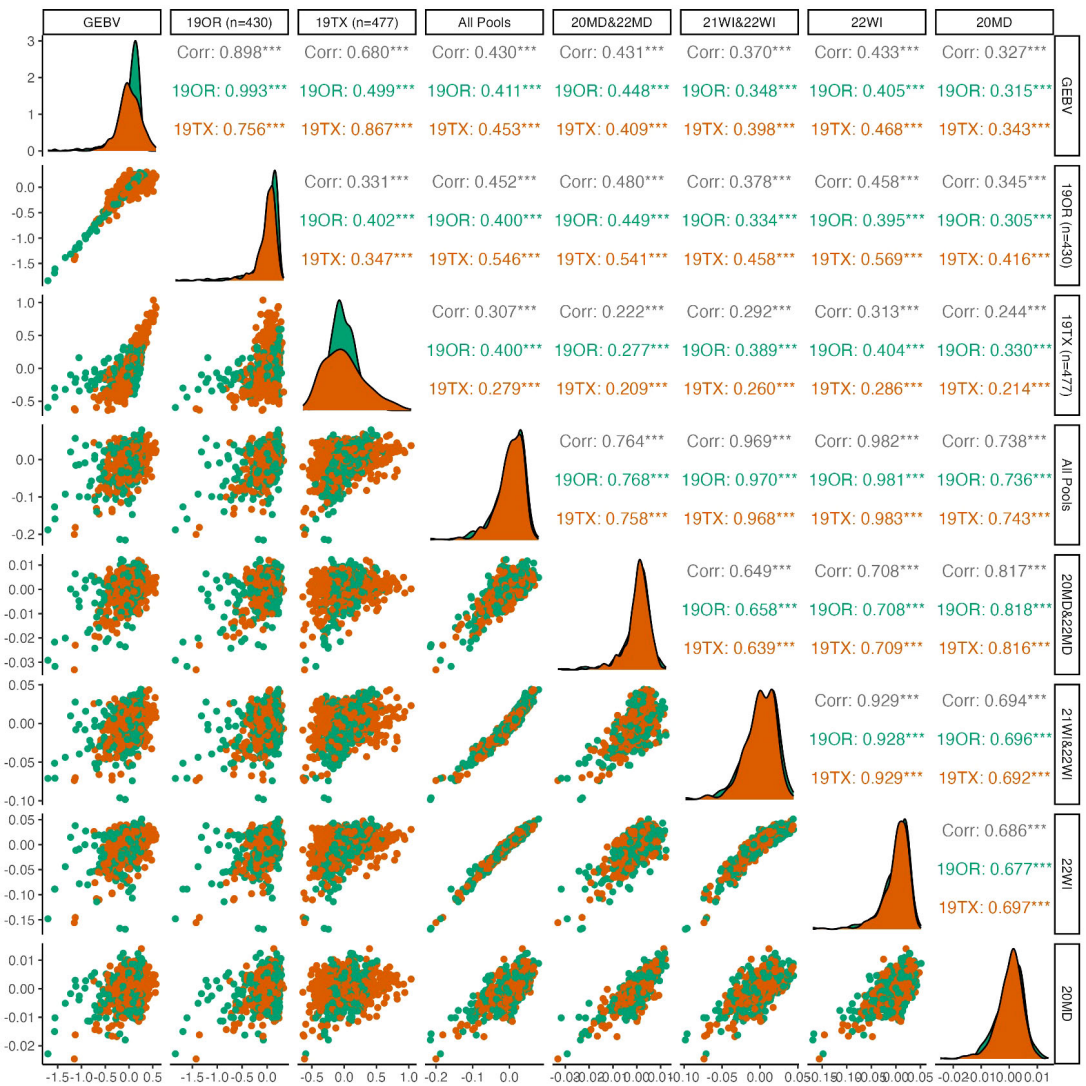
Pairwise plots and correlations of pod dehiscence. The genotypically estimate breeding values (GEBV) represents the ‘true’ breeding values predicted by using all 19OR and 19TX field data and the remainder of the columns/rows represent subsets of data attempting to predict the GEBVs. Point clouds (below diagonal), histograms (diagonal), and correlations (above diagonal) are divided by site-year with Oregon in green and Texas in orange. Asterisk indicate levels of significance, with *** indicating p<0.001, ** indicating p<0.01, and * indicating p<0.05.

**Figure 3:**
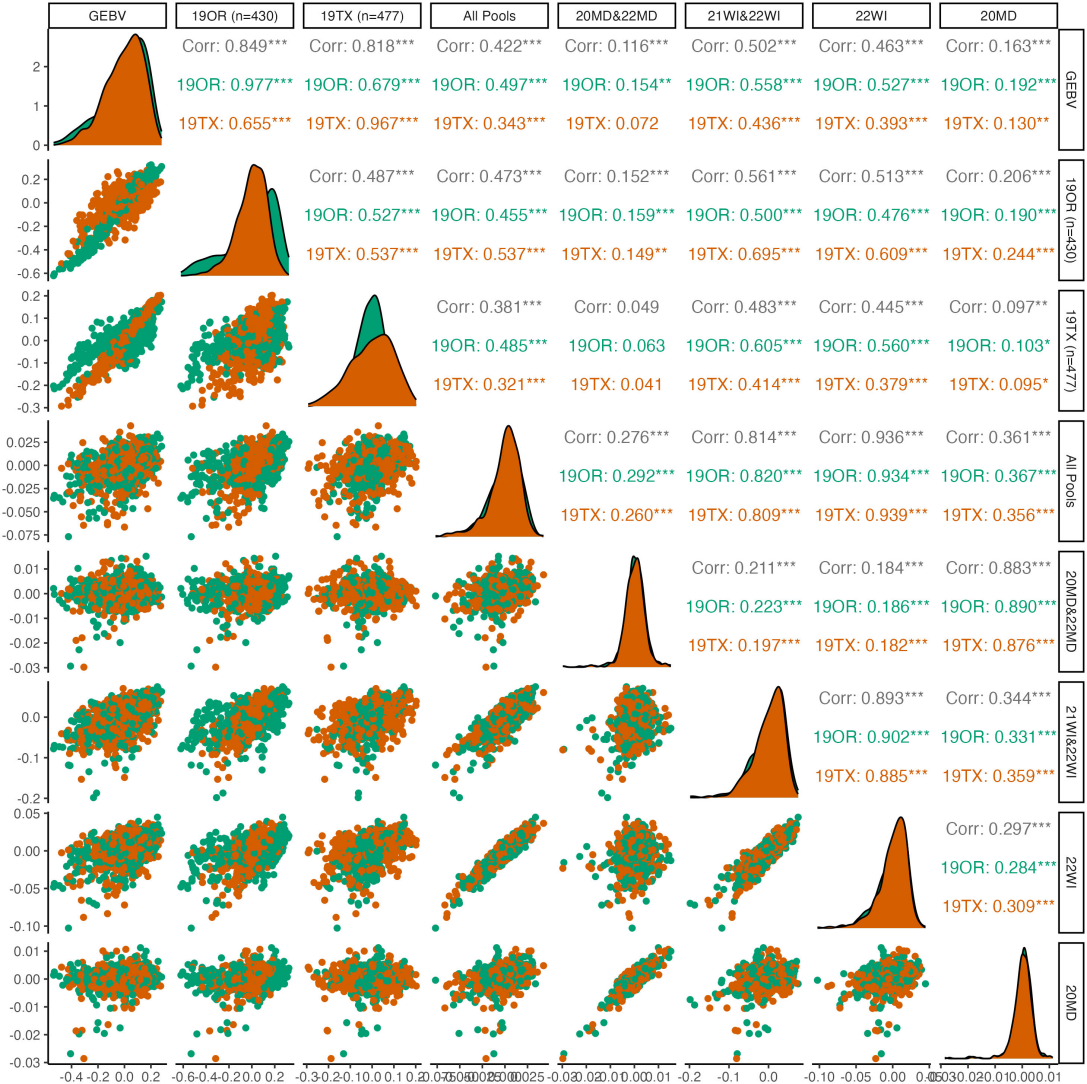
Pairwise plots and correlations of seed dormancy. The genotypically estimate breeding values (GEBV) represents the ‘true’ breeding values predicted by using all 19OR and 19TX field data and the remainder of the columns/rows represent subsets of data attempting to predict the GEBVs. Point clouds (below diagonal), histograms (diagonal), and correlations (above diagonal) are divided by site-year with Oregon in green and Texas in orange. Asterisk indicate levels of significance, with *** indicating p<0.001, ** indicating p<0.01, and * indicating p<0.05.

Genomic prediction indicated consistently (p<0.01) positive predictive ability between environments for pod dehiscence, with mixed results for prediction of seed dormancy (**Figures 2**, **3**). For pod dehiscence, predictive ability of the DNA pools for 19OR (r=0.411; S.E. = 0.044) and 19TX (r=0.453; S.E. = 0.040) was lower than predictive ability of the model where OR was used to predict 19TX (r=0.756; S.E. = 0.030). However, the pooled DNA samples had no statistically significant difference from the model using 19TX to predict 19OR (r=0.499; S.E. = 0.041). For pooled DNA sample subsets, the Maryland pooled DNA samples had marginally greater pod dehiscence predictive ability relative to Wisconsin pooled DNA samples (r=0.431; S.E. = 0.030 vs. r=0.370; S.E. = 0.030). Many fluctuations in predictive ability are due to the skewed distribution of pod dehiscence GEBVs at 19OR.

For seed dormancy, predictive ability of the complete set of DNA pools for 19OR (r=0.497; S.E=0.040) and 19TX (r=0.343; S.E. = 0.041) was lower than predictive ability between the two environments (19OR predicting 19TX: r=0.655; S.E. = 0.034; 19TX predicting 19OR: r=0.679; S.E. = 0.035). Within the seed dormancy predictions, the Maryland pooled DNA samples had exceptionally poor predictive ability (r=0.116; S.E. = 0.033) relative to the Wisconsin pooled DNA samples (r=0.502; S.E. = 0.028). The correlation between 20MD and 22MD pooled DNA sample predictions was also weaker (r=0.345; S.E. = 0.031) relative to the correlations between 21WI and 22WI pooled DNA sample predictions (r=0.647; S.E. = 0.025).

**Figure 4:**
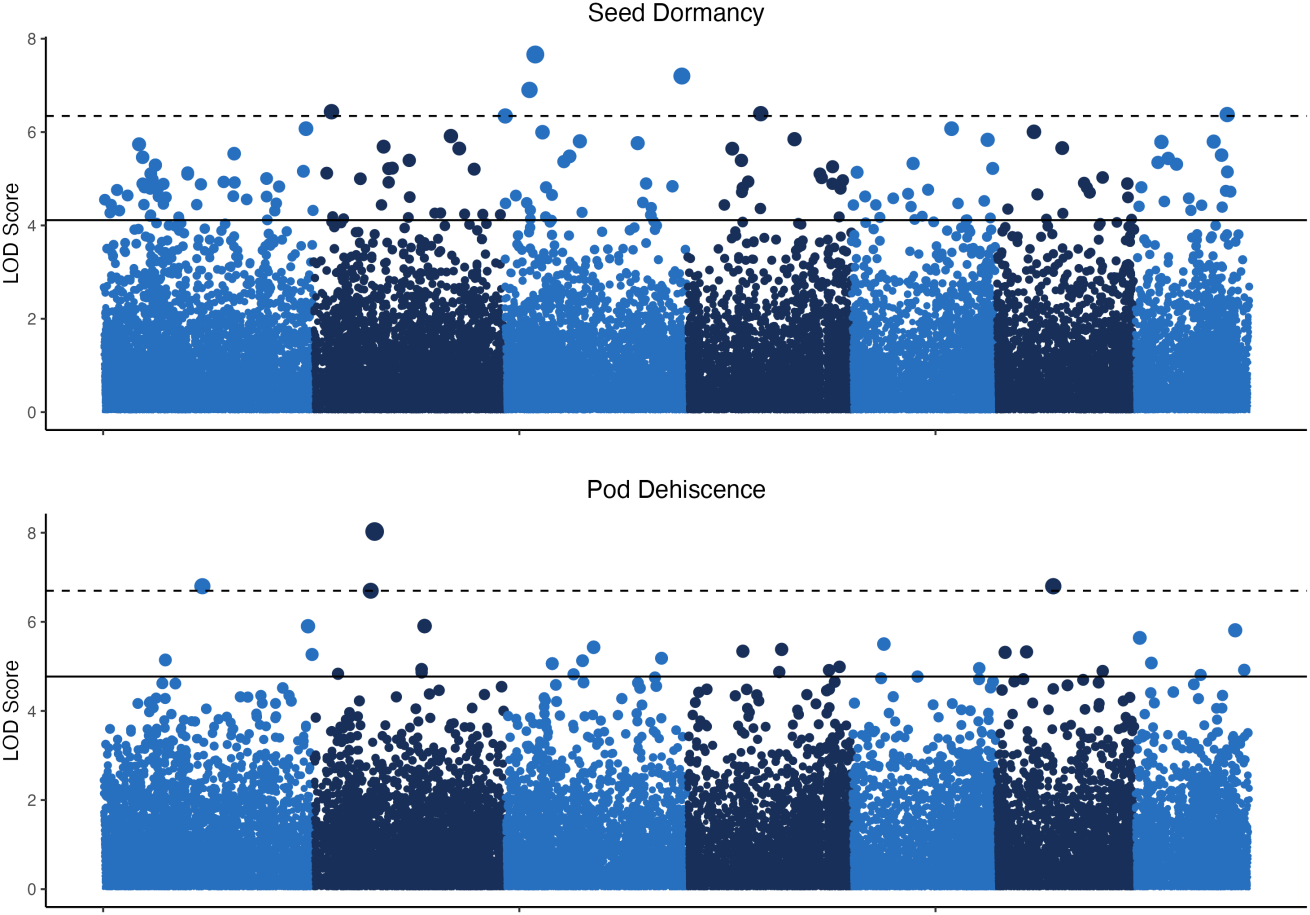
Manhattan plots for seed dormancy and pod dehiscence based on 24 pooled DNA samples. The y-axis indicates the logarithm of odds (LOD) scores of p-values for individual SNPs and the horizontal lines indicate the p–value equal to the false discovery rates at q<0.05 (solid line) and q<0.01 (dashed line).

**Figure 5:**
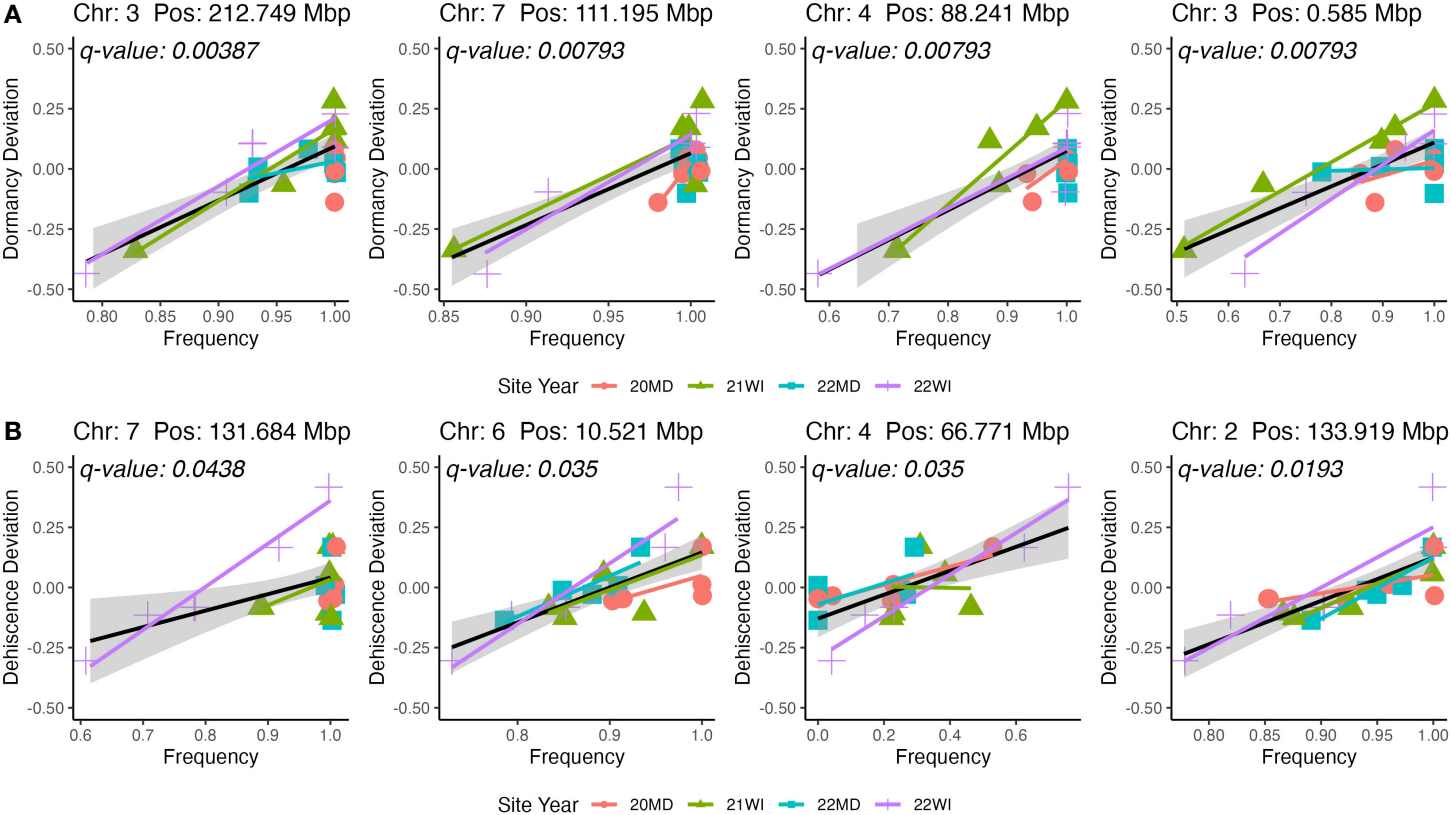
Representations of marker effects for the four highest significance QTL for seed dormancy **(A)** and pod dehiscence **(B)**. Only beneficial QTL were included in the figure for pod dehiscence. The mean site performance at each site-year was subtracted from each set of DNA pools. The deviation of each pooled DNA sample from the mean site-year performance is plotted on the y-axis. The x-axis is the allele frequency for the QTL. Colors and point shapes represent different site-years.

The GWAS was based on 24 pooled DNA samples resulted in six significant QTL for seed dormancy and four significant QTL for pod dehiscence (**Figure 4**). Unfortunately, all significant pod dehiscence regions were minor alleles which increased the rate of pod dehiscence. Therefore, these are unlikely to be useful for breeding. The next four most likely regions which could reduce pod dehiscence had q-values of 0.019 (Chromosome 2: Position 133.919 Mbp), 0.035 (Chromosome 4: Position 66.771 Mbp), 0.035 (Chromosome 6: 10.521 Mbp) and 0.438 (Chromosome 7: Position 131.684 Mbp; **Figure 5**). The last allele (Chromosome 7) was significant only due to a strong effect within a single environment (22WI; **Figure 5**). Broadly, the pooled DNA GWAS samples QTLs tended to be in SNPs with a high rate (>20%) of missing allele calls in the individual GWAS panel (**Supplementary Table 1**).

**Figure 6:**
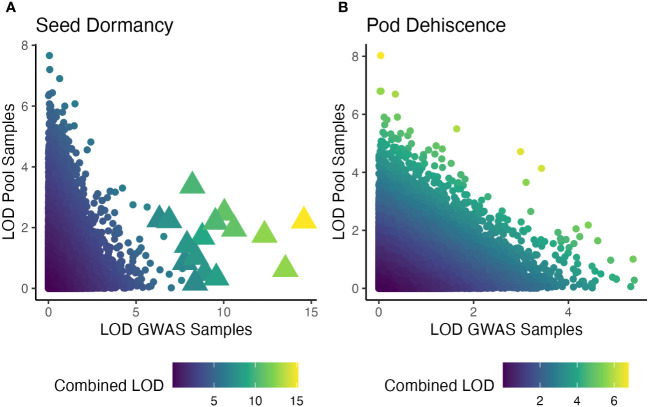
Significance of statistical tests for two traits and for two GWAS analysis presented as logarithm of odds (LOD) score. The x-axis is an individual-based GWAS using two site-years (Oregon and Texas in 2019). The y-axis is a pooled DNA sample based GWAS using four site-years (Maryland in 2020 and 2022; Wisconsin in 2021 and 2022). Color is the LOD score of the combined p-values using the Fisher’s combined probability test. Note unequal color scales across seed dormancy **(A)** and pod dehiscence **(B)**. Significant markers, based on a false-discovery rate of q<0.01 are large triangles, while insignificant markers are small circles.

When the individual-based GWAS and pooled DNA sample-based GWAS were combined, many seed dormancy regions were significant at q<0.01, but no pod dehiscence regions had significant effects (**Figure 6**). The significant seed dormancy regions were largely due to strong p-values within the individual GWAS panel and moderate p-values within the pooled DNA sample GWAS. Two pod dehiscence regions had q-value<0.05 and were due to strong p-values across both datasets.

## Discussion

The DNA pools in this study were sufficient to train genomic prediction models which accurately predicted performance across multiple environments (**Figures 2**, **3**). This result is evidence of a lack of rank-change genotype-by-environment (GxE) variation for these two important traits. Genetic variance of seed dormancy (Kissing Kucek et al., 2020a) and pod dehiscence is larger than GxE variance. Therefore, selection at one environment can improve the trait across all environments in this set. This result is useful since the environments span the breadth of the continental United States.

The predictive ability of pooled DNA samples tended to be lower (0.63 vs 0.43 for pod dehiscence and 0.66 vs 0.42 for seed dormancy) than the dataset which used individual genotypes. However, pooled DNA sample-based predictions were positive and sufficient to make genetic gain at a fraction of the cost of individual DNA sequencing. The pooled DNA samples had comparable predictive ability despite greater genetic distance from the validation population, less phenotypic information, and less sequencing investment. Specifically, the 19OR and 19TX environments shared a common set of 40 half-sib families which were replicated across both environments. By contrast, the pooled DNA samples used individuals which were one to three generations separated from the individual GWAS panel. Similarly, the filtered individual GWAS panel required 3.2 billion reads total (869 individuals with 3.7 million reads each), which resulted in a read depth of 4.1 per site per sample in the final dataset. By contrast, the 24 pooled DNA samples required 504 million reads total (21 million raw reads each), which resulted in a read depth of 53.8 in the final dataset. Sample handling and DNA extraction of hundreds of individuals required more resources relative to the pooled DNA samples which simply combined evenly sized leaf tissue prior to homogenization and DNA extraction. In this study, individual field data had already been collected, but a future study would only need to dedicate enough resources to phenotyping to determine if an individual is an outlier (top or bottom 25%, for example) for the trait of interest. The aggregated phenotypic values used for prediction highlight the degree in which coarse phenotyping is sufficient to train pooled DNA genomic prediction (**Figure 2**). Prior simulations indicate that numerically large DNA pools can tolerate a large degree of phenotyping error due to these coarse thresholds (Tilhou and Casler, 2021). Overall, this study provides further evidence that pooled DNA sequencing is a promising method to reduce the resources required for genomic breeding methods.

The Maryland pooled DNA samples poorly predicted seed dormancy at either 19OR or 19TX (**Figure 3**). The Maryland pooled DNA sample seed dormancy GEBVs also had a weaker correlation to Wisconsin pooled DNA sample seed dormancy GEBVs when compared to the pod dehiscence GEBVs at the two environments (0.649 vs 0.211; **Figures 2**, **3**). Since the Maryland pooled DNA samples were able to adequately predict pod dehiscence, the pool sample sizes and genetic relationships between germplasm at the two environments must be adequate. There are three possible causes for this observation. First, the major alleles reducing seed dormancy in this breeding population may be ineffective in Maryland for an unknown reason. Alternatively, the poor prediction may be due to the strongly skewed distributions of seed dormancy in Maryland (Kissing Kucek et al., 2020a). The small number of outliers could be causing excess variation in the data. Last, there may simply be reduced heritability in Maryland (**Figure 3**), since predictions based on individual Maryland DNA pools (20MD vs. MD22) were weakly correlated (0.345) relative to the correlation between Wisconsin (21WI vs. 22WI) DNA pool predictions (0.647). Kissing Kucek et al. (2020a) reported that growing environments accounted for 28% of trait variance but that the relationship between growing season conditions and seed dormancy were highly variable. This study helps confirm this result, but the pooled DNA samples are not from enough environments to allow speculation on the mechanism of the GxE variance impacting the Maryland environments.

Genomic prediction uses data spread throughout the genome and therefore requires a lower quantity of genomic data relative to what is required for determining significance at a single SNP site for association studies. If this study was repeated, greater sequencing depth and ideally a greater number of individuals would be used during pooled DNA sample construction to increase the ability to confidently distinguish QTL. For example, the major QTL for seed dormancy reported in Tilhou et al. (2023) had a mean read depth of only 11 in the pooled DNA samples of the current study, which was not sufficient for inclusion in the analysis. Similarly, GWAS analyses which used subsets of pooled DNA samples (single environments) did not result in clear differentiation of potential QTL (data not presented). A major downside of analyzing a small number of pooled DNA samples is the inability to identify the issues which reduce statistical power. The numerically small DNA pools (n=29-74) may contain population structure which creates an excess of false positives. Alternatively, insufficient read depth for the DNA pools could prevent differentiation of significant QTL in single environments. Despite this, the combined set of 24 pooled DNA samples were sufficient to detect QTL (**Figure 4**).

The initial plan for analysis was to perform a GWAS analysis for each trait using only contrasting pooled DNA samples for each trait (pod dehiscence or dormant seed) and random interquartile pooled DNA samples for each environment. However, the mean performance of pooled DNA samples were correlated (**Figure 1**) so all pooled DNA samples were used in the analysis of each trait to maximize the information available. The potential positive phenotypic correlation (0.37) between pod dehiscence and seed dormancy aligns with the phenotypic correlation reported in Tilhou et al. (0.29; 2023). In hindsight, slightly increasing the number of DNA pools and constructing DNA pools to minimize the within-pool multi-trait phenotypic variance at each environment may have been a superior strategy. This strategy was evaluated in two livestock studies and was found to have equivalent predictive ability relative to individual samples in genomic prediction (Baller et al., 2020, Baller et al., 2022). For the analysis presented in this study, a more complex pool construction strategy may have increased the proportion of information available to compensate for the limited population sizes at each environment (n=115-287).

The current study was an attempt to both validate the previously published seed dormancy QTL and potentially locate a major QTL for reducing pod dehiscence. Strictly speaking, we failed at both of those goals. In theory, replicating a GWAS using additional environments will improve the ability to detect significant QTL. However, in the current study there was nearly no overlap between the QTL results for both traits (**Figure 6**). All of the significant QTL from the pooled DNA sample GWAS were insignificant when combined with the individual GWAS panel p-values. The two GWAS studies used different environments, different breeding populations, and different sequencing runs. Any of these factors could have contributed to disagreements between results. Despite these issues, the QTL from each GWAS and the combined GWAS appear to have equal likelihood of being valid and promising QTL will be further explored (**Supplementary Table 1**).

Although the major seed dormancy QTL from Tilhou et al. (2023) was not directly validated in the current study, a parallel project identified a haplotype in chromosome 1 which strongly reduces seed dormancy (data not presented; in progress). This marker will be applied to the hairy vetch program beginning in 2024. Interestingly, the closest significant marker in the current study (Chromosome 1: 63631572: **Supplementary Table 1**) decreased in frequency over the course of the breeding program (19OR: 0.87; 19TX: 0.90; 20MD: 0.77; 21WI: 0.70; 22MD 0.57; 22WI 0.57). Therefore, there is selection in this region and this SNP appears to be in linkage with the true QTL in the region. This degree of breeding progress is promising validation that there is a significant QTL in this region since this signature of selection occurred prior to identification of the QTL.

Intentional selection pressure to reduce seed dormancy varied by environment in the hairy vetch breeding program due to simultaneous selection for many traits (spring vigor, fall vigor, emergence, pod dehiscence, flowering time, seed yield). The 21WI and 22WI cohorts received more intense selection pressure for reduced seed dormancy by advancing the lowest 1% of half-sibling families from the entire breeding program based on an animal model. The 20MD and 22MD cohorts were the result of weaker selection pressure for seed dormancy, truncating families with seed dormancy above the overall mean. Incidental selection against dormant seed also occurs within each family during every breeding cycle since dormant seeds immediately discarded from the program. This incidental selection occurs less for pod dehiscence and could explain the differences between the two traits in these studies. Steady incidental selection against dormant seed could more rapidly increase initially rare seed dormancy QTL to the frequency where it could be detected, while pod dehiscence receives a lower weight in selection indices relative to other traits.

In the current breeding population, it appears that pod dehiscence is highly polygenic. The strongest pod dehiscence QTL in the pooled DNA samples were rare alleles which increased pod dehiscence (**Figure 3**). Therefore, it is possible that multiple overlapping or epistatic QTL may need to be eliminated from the population. There is an intriguing result in one of the weak (q-value<0.05) dehiscence QTL. Similar to the seed dormancy QTL in chromosome 1, there is a pod dehiscence QTL (Chromosome 4: 66771196; **Supplementary Table 1**; **Figure 4**; panel B) which decreases across the sampled years of the breeding program (19OR: 0.46; 19TX: 0.46; 20MD: 0.19; 21WI: 0.29; 22MD 0.16; 22WI 0.33). The beneficial (low pod dehiscence) allele now appears to be the major allele in the program. Therefore, marker assisted selection is not cost effective for this QTL but, it implies that ongoing non-molecular breeding efforts may be effective in reducing dehiscence in this breeding program.

## Data availability statement

Allele frequencies and phenotypic data are available on Dryad Digital Repository (https://doi.org/10.5061/dryad.s4mw6m9d6).

## Author contributions

NT: Conceptualization, Data curation, Formal analysis, Investigation, Methodology, Writing – original draft, Writing – review & editing. LK: Conceptualization, Data curation, Funding acquisition, Investigation, Methodology, Supervision, Writing – review & editing. BC: Data curation, Funding acquisition, Supervision, Writing – review & editing. JD: Data curation, Funding acquisition, Supervision, Writing – review & editing. JE: Data curation, Funding acquisition, Supervision, Writing – review & editing. SA: Data curation, Writing – review & editing. JR: Data curation, Methodology, Supervision, Writing – review & editing. SB: Data curation, Supervision, Writing – review & editing. SM: Data curation, Funding acquisition, Supervision, Writing – review & editing. MM: Data curation, Funding acquisition, Supervision, Writing – review & editing. RH: Data curation, Funding acquisition, Supervision, Writing – review & editing. HR: Conceptualization, Data curation, Funding acquisition, Investigation, Project administration, Supervision, Writing – review & editing.

## Supplementary material

Supplementary Figure 1 Scatter plot and smoothed (loess) lines indicating the relationship between allele frequencies across four pairs of technical replicates. The colors indicate the read depth of each marker. Hard seed refers to seed dormancy and shatter refers to pod dehiscence.

Supplementary Figure 2 Representations of QTLs with potential for breeding progress Fisher q-value for dehiscence <0.05. Scatter plots (top) indicate the QTL frequency in pooled DNA samples (x-axis) and the deviation from site-year means for the traits of interest (y-axis; seed dormancy or pod dehiscence). Boxplots (below) represent individual data across two site-years (19OR and 19TX) based on centered (-1, 0, 1) allele dosage. Logarithm of odds (LOD) scores in red are for the pooled DNA GWAS (top) or individual-based GWAS (bottom). Significant QTL were determined from combining p-values using Fisher’s combined probability test and multiple testing was corrected to control the false discovery rate. Q-value threshold for seed dormancy <0.0001. Q-value threshold for pod dehiscence <0.05.

Supplementary Table 1 Details on major QTL of interest for seed dormancy and dehiscence. Seed dormancy QTL are included if the q<0.0001. Pod dehiscence QTL are included if q<0.05 and the minor allele confers lower pod dehiscence rates. Allele differences indicate the change in allele frequencies between the individual DNA samples (from 2019) and pooled DNA samples (2020-2022).
