# Supplementary figures and images for: Pooled DNA sequencing in hairy vetch (Vicia villosa Roth) reveals QTL for seed dormancy but not pod dehiscence
Source: Front Plant Sci. 2024 Apr 4;15:1384596. doi: 10.3389/fpls.2024.1384596 (PMC11024373; doi:10.3389/fpls.2024.1384596)

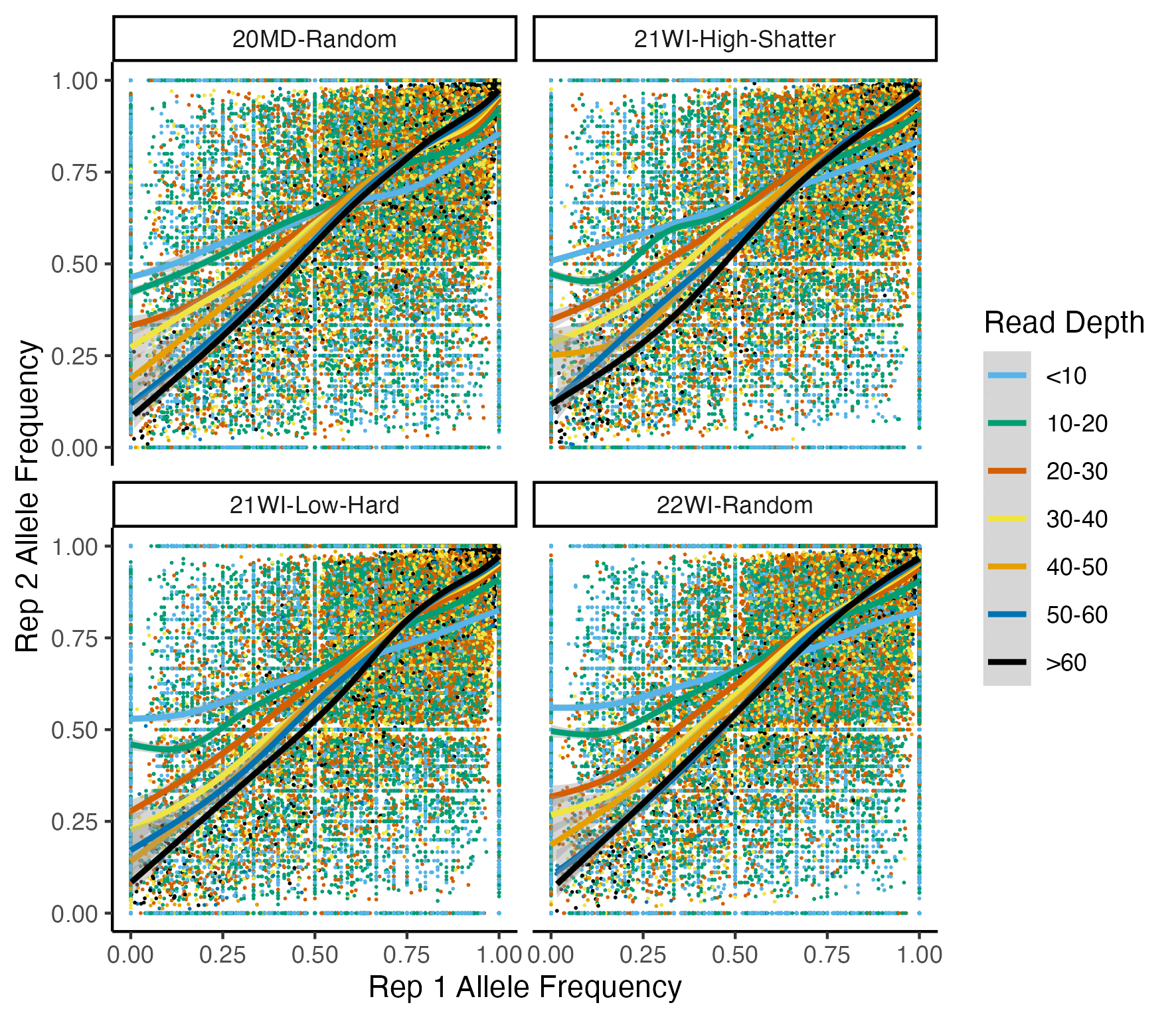

Supplement: Supplementary Figure 1 — Scatter plot and smoothed (loess) lines indicating the relationship between allele frequencies across four pairs of technical replicates. The colors indicate the read depth of each marker. Hard seed refers to seed dormancy and shatter refers to pod dehiscence. [file Image_1.tiff]

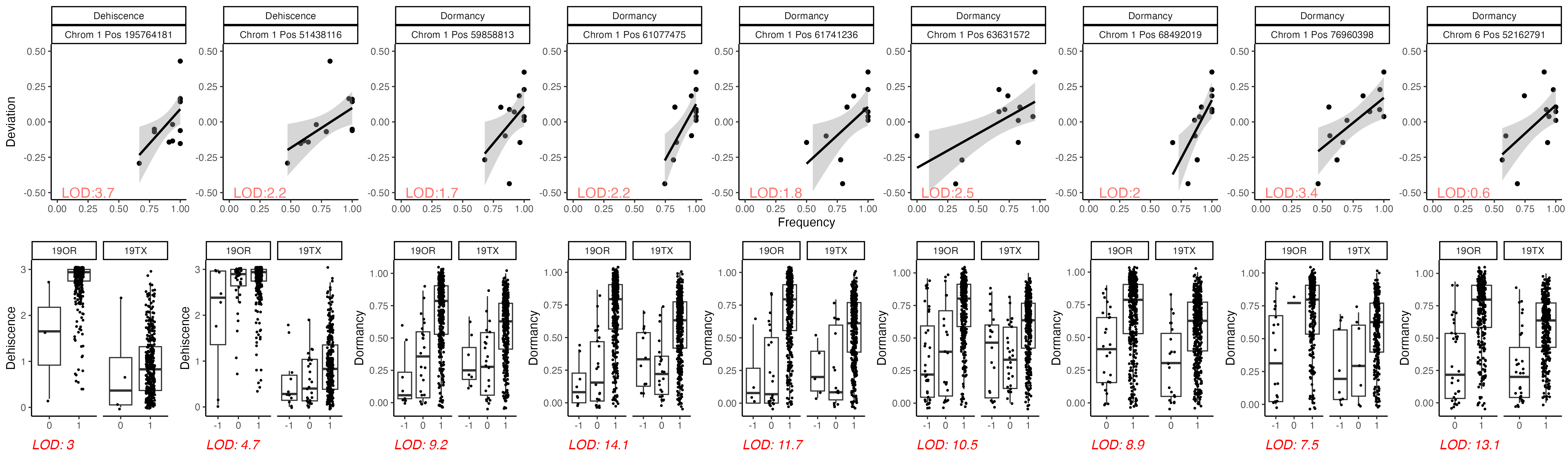

Supplement: Supplementary Figure 2 — Representations of QTLs with potential for breeding progress Fisher q-value for dehiscence <0.05. Scatter plots (top) indicate the QTL frequency in pooled DNA samples (x-axis) and the deviation from site-year means for the traits of interest (y-axis; seed dormancy or pod dehiscence). Boxplots (below) represent individual data across two site-years (19OR and 19TX) based on centered (-1, 0, 1) allele dosage. Logarithm of odds (LOD) scores in red are for the pooled DNA GWAS (top) or individual-based GWAS (bottom). Significant QTL were determined from combining p-values using Fisher’s combined probability test and multiple testing was corrected to control the false discovery rate. Q-value threshold for seed dormancy <0.0001. Q-value threshold for pod dehiscence <0.05. [file Image_2.jpg]
