# Supplementary material for: Pooled DNA sequencing in hairy vetch (Vicia villosa Roth) reveals QTL for seed dormancy but not pod dehiscence
Source: Front Plant Sci. 2024 Apr 4;15:1384596. doi: 10.3389/fpls.2024.1384596 (PMC11024373; doi:10.3389/fpls.2024.1384596)
Supplement: Supplementary Table 1 — Details on major QTL of interest for seed dormancy and dehiscence. Seed dormancy QTL are included if the q<0.0001. Pod dehiscence QTL are included if q<0.05 and the minor allele confers lower pod dehiscence rates. Allele differences indicate the change in allele frequencies between the individual DNA samples (from 2019) and pooled DNA samples (2020-2022). [file Table_1.docx]

Supplementary table 1: Details on major QTL of interest for seed dormancy and dehiscence. Seed dormancy QTL are included if the q<0.0001. Pod dehiscence QTL are included if q<0.05 and the minor allele confers lower pod dehiscence rates. Allele differences indicate the change in allele frequencies between the individual DNA samples (from 2019) and pooled DNA samples (2020-2022)

| Chr. | Pos. | Pooled  GWAS p-value | Individual GWAS p-value | Fisher  p-value | Fisher  q-value | Trait | Individual GWAS Missing (%) | DNA Pool Read Depth | Reference Allele | Alternate Allele | Allele difference | Significance Source |
| --- | --- | --- | --- | --- | --- | --- | --- | --- | --- | --- | --- | --- |
| 2 | 22129240 | 3.66E-07 | - |  |  | Dormancy | 0.20 | 32.3 | C | A | 0.02 | Pools |
| 3 | 585434 | 4.52E-07 | - |  |  | Dormancy | 0.35 | 35.8 | C | T | -0.07 | Pools |
| 3 | 29695626 | 1.25E-07 | - |  |  | Dormancy | 0.18 | 45.4 | C | T | 0.01 | Pools |
| 3 | 36661939 | 2.18E-08 | - |  |  | Dormancy | 0.11 | 32.5 | T | C | -0.03 | Pools |
| 3 | 212749438 | 6.30E-08 | - |  |  | Dormancy | 0.06 | 60.7 | G | C | 0.00 | Pools |
| 4 | 88240616 | 4.03E-07 | - |  |  | Dormancy | 0.13 | 39.3 | G | A | 0.05 | Pools |
| 7 | 111195178 | 4.21E-07 | - |  |  | Dormancy | 0.18 | 33.0 | T | C | -0.01 | Pools |
| 2 | 133919438 | 1.24E-06 | - |  |  | Dehiscence | 0.23 | 35.3 | C | T | 0.04 | Pools |
| 4 | 66771196 | 4.57E-06 | - |  |  | Dehiscence | 0.43 | 32.3 | A | T | -0.22 | Pools |
| 6 | 10521433 | 4.85E-06 | - |  |  | Dehiscence | 0.24 | 42.6 | T | A | -0.03 | Pools |
| 7 | 131683540 | 1.20E-05 | - |  |  | Dehiscence | 0.37 | 34.1 | C | A | -0.03 | Pools |
| 1 | 61077475 | 6.00E-03 | 7.21E-15 | 1.67E-15 | 2.06E-10 | Dormancy | 0.05 | 59.3 | T | C | -0.03 | Dual |
| 6 | 52162791 | 2.35E-01 | 8.31E-14 | 6.36E-13 | 7.80E-08 | Dormancy | 0.21 | 36.4 | G | A | -0.13 | Dual |
| 1 | 61741236 | 1.76E-02 | 1.90E-12 | 1.07E-12 | 1.31E-07 | Dormancy | 0.08 | 43.5 | G | T | -0.06 | Dual |
| 1 | 63631572 | 3.31E-03 | 3.08E-11 | 3.15E-12 | 3.86E-07 | Dormancy | 0.16 | 38.7 | G | A | -0.23 | Dual |
| 1 | 59858813 | 2.04E-02 | 5.95E-10 | 3.18E-10 | 3.90E-05 | Dormancy | 0.16 | 66.9 | C | T | -0.04 | Dual |
| 1 | 76960398 | 4.23E-04 | 3.08E-08 | 3.40E-10 | 4.18E-05 | Dormancy | 0.37 | 36.9 | T | A | -0.14 | Dual |
| 1 | 68492019 | 1.11E-02 | 1.27E-09 | 3.66E-10 | 4.50E-05 | Dormancy | 0.02 | 64.0 | T | C | -0.05 | Dual |
| 1 | 51438116 | 6.56E-03 | 2.11E-05 | 2.32E-06 | 3.17E-02 | Dehiscence | 0.09 | 57.2 | C | T | -0.12 | Dual |
| 1 | 195764181 | 2.21E-04 | 9.18E-04 | 3.33E-06 | 4.10E-02 | Dehiscence | 0.05 | 41.2 | G | A | -0.05 | Dual |
